# Potential Link between the Sphingosine-1-Phosphate (S1P) System and Defective Alveolar Macrophage Phagocytic Function in Chronic Obstructive Pulmonary Disease (COPD)

**DOI:** 10.1371/journal.pone.0122771

**Published:** 2015-10-20

**Authors:** Jameel Barnawi, Hai Tran, Hubertus Jersmann, Stuart Pitson, Eugene Roscioli, Greg Hodge, Robyn Meech, Rainer Haberberger, Sandra Hodge

**Affiliations:** 1 Lung Research, Hanson Institute, Adelaide, Australia; 2 Dept of Medicine, University of Adelaide, Adelaide, Australia; 3 Dept Medical Laboratory Technology, University of Tabuk, Tabuk, Saudi Arabia; 4 Centre for Cancer Biology, University of South Australia, Adelaide, Australia and SA Pathology, Adelaide, Australia; 5 Department of Clinical Pharmacology, Flinders University, Adelaide, Australia; 6 Centre for Neuroscience, Anatomy & Histology, Flinders University, Adelaide, Australia; University of Tübingen, GERMANY

## Abstract

**Introduction:**

We previously reported that alveolar macrophages from patients with chronic obstructive pulmonary disease (COPD) are defective in their ability to phagocytose apoptotic cells, with a similar defect in response to cigarette smoke. The exact mechanisms for this defect are unknown. Sphingolipids including ceramide, sphingosine and sphingosine-1-phosphate (S1P) are involved in diverse cellular processes and we hypothesised that a comprehensive analysis of this system in alveolar macrophages in COPD may help to delineate the reasons for defective phagocytic function.

**Methods:**

We compared mRNA expression of sphingosine kinases (*SPHK1/2*), S1P receptors *(S1PR1-5)* and S1P-degrading enzymes (*SGPP1*, *SGPP2*, *SGPL1*) in bronchoalveolar lavage-derived alveolar macrophages from 10 healthy controls, 7 healthy smokers and 20 COPD patients (10 current- and 10 ex-smokers) using Real-Time PCR. Phagocytosis of apoptotic cells was investigated using flow cytometry. Functional associations were assessed between sphingosine signalling system components and alveolar macrophage phagocytic ability in COPD. To elucidate functional effects of increased *S1PR5* on macrophage phagocytic ability, we performed the phagocytosis assay in the presence of varying concentrations of suramin, an antagonist of *S1PR3* and *S1PR5*. The effects of cigarette smoking on the S1P system were investigated using a THP-1 macrophage cell line model.

**Results:**

We found significant increases in *SPHK1/2* (3.4- and 2.1-fold increases respectively), *S1PR2* and *5* (4.3- and 14.6-fold increases respectively), and *SGPL1* (4.5-fold increase) in COPD vs. controls. *S1PR5* and *SGPL1* expression was unaffected by smoking status, suggesting a COPD “disease effect” rather than smoke effect *per se*. Significant associations were noted between *S1PR5* and both lung function and phagocytosis. Cigarette smoke extract significantly increased mRNA expression of *SPHK1*, *SPHK2*, *S1PR2* and *S1PR5* by THP-1 macrophages, confirming the results in patient-derived macrophages. Antagonising *SIPR5* significantly improved phagocytosis.

**Conclusion:**

Our results suggest a potential link between the S1P signalling system and defective macrophage phagocytic function in COPD and advise therapeutic targets.

## Introduction

Chronic obstructive pulmonary disease (COPD) is a major cause of morbidity and mortality worldwide. Cigarette smoking is a major cause of COPD, yet despite the huge campaign that encourages smoking cessation worldwide, the smoking incidence is only slowly decreasing in developed countries and still increasing in developing countries. COPD is an incurable disease and currently available treatments are largely ineffective [1,2]. There is therefore an urgent need for further understanding the pathophysiology of COPD to advise effective new therapies.

In previous studies our group has shown that alveolar macrophages from COPD patients are defective in their ability to phagocytose apoptotic cells despite smoking cessation (defective efferocytosis) [3–7]. We and others have shown that if these apoptotic cells are not cleared effectively by alveolar macrophages, they may undergo secondary necrosis which can further promote the inflammation in the lung [8,9]. We have also shown that both alveolar macrophages and monocyte-derived macrophages from COPD patients are impaired in their ability to phagocytose bacteria which might potentially contribute to bacterial colonization in COPD [10]. Numerous molecules have been identified as possible contributors to these macrophage phenomena in COPD [11], but the exact mechanism is yet unknown.

Sphingolipid metabolites including ceramide, sphingosine andsphingosine-1-phosphate (S1P) are involved in diverse cellular processes. Phosphorylation of sphinosine by the sphingosine kinases (*SPHK*s) results in the production of S1P, while its acetylation by ceramide synthase produces ceramide, and then sphingomyelin after sphingomyelin synthase catalysed coupling to phosphocholine. Structural changes in these molecules have been proven to result in modulation of cellular function [11]. Many sphingolipid molecules have been linked to inflammatory lung diseases and used as potential therapeutic targets, such as acid sphingomyelinase in acute lung injury [12], neutral sphingomyelinase-2 in COPD [13], and ceramide in cystic fibrosis [14].

We have previously shown that human lung tissue comprises a complex expression profile for the individual components of the S1P signalling system including synthesising enzymes, receptors and degrading enzymes [15]. In COPD we found correlations between mRNA expression levels of several receptors and enzymes involved in the S1P signalling system in the lung suggesting common regulatory mechanisms. However, we did not assess individual lung cells such as alveolar macrophages, which are likely to be of importance in COPD. S1P is one of the most important sphingolipid molecules and its role in immune cell function has been shown in many studies [16,17]. It has been shown to be involved in a multitude of cellular signalling pathways and responses, such as proliferation, survival and growth [18] and exerts its function through five G-protein coupled receptors (*S1PR1-5*) [19].

The alveolar macrophage is a very interesting target for our investigations since it has been shown that S1P regulates macrophage function, phagosome maturation and migration [20]. The phagocytic function of macrophages has also been shown to be modulated by the sphingosine system; for example, it has been shown that phagocytosis of *M*.*tuberculosis* bacteria depends on *SPHK1* [21]. S1P which has been implicated in macrophage actin assembly and phagosome function [22] was shown to improve phagocytosis of *Cryptococcus neoformans* when exogenously added to wild-type alveolar macrophages [23]. Furthermore, ceramide, the precursor of S1P, has an opposing role to S1P and has been found to decrease macrophage efferocytosis in COPD [24]. Taken together, these studies suggest that defective efferocytosis in alveolar macrophages in COPD may be associated with the S1P system. Despite the numerous studies on this system and its role in inflammation and diseases, to the best of our knowledge, there have been no comprehensive studies of the S1P system in macrophages from healthy controls, or of the role of this system in the defective macrophage function in COPD. In this study, we compared the expression of components of the S1P-signalling system in alveolar macrophages from healthy control volunteers and COPD patients. We determined the expression profiles of *SPHK1*, *SPHK2*, receptors (*S1PR1*-5) and the S1P-degrading enzymes, sphingosine-1-phosphate phosphatase 1 and 2 (*SGPP1* and *SGPP2*) and sphingosine-1-phosphate lyase 1 (*SGPL1*) and their correlation with the ability of alveolar macrophages to phagocytose apoptotic cells. We further investigated functional consequences of smoking on the expression profile of the S1P system in current- or ex-smoking COPD subjects and by using an *in vitro* THP-1 macrophage cell line model.

## Material and Methods

### Categorization of patients

This study was approved by the Royal Adelaide Hospital Ethics Committee (Adelaide, Australia) and informed written consent was obtained for each subject in this study. Patients were categorized based on gender, smoking status, age, the presence of lung cancer and lung function (Table 1). The diagnosis of COPD was carried out according to the Global Initiative for Chronic Obstructive Lung Disease (GOLD) standards. Any subject who had ceased smoking within the previous 6 months was included in the ‘current smoker’ groups. Exclusion criteria included diagnosis of other inflammatory lung diseases, blood malignancy and current infection.

**Table 1:** Patient demographics.

	Controls	Control Cur-Smoker	COPD	COPD Cur-smoker	COPD Ex-Smoker
Number (n)	10	7	20	10	10
Age (yr)	56.5(26–71)	36(28–46)	66.5(42–86)	60(42–75)	74(49–86)
Smoking(cur/ex/no)	0/6/4	0/0/7	10/10/0	10/0/0	0/10/0
Gender (M/F)	6/4	2/5	14/6	5/5	9/1
Lung Cancer (y/n)	0/10	0/7	10/10	4/6	6/4
Type of Lung Cancer (NSCLC/SCLC)	0/0	0/0	10/0	4/0	6/0
Type of NSCLC (adenos /squa/ larg)	0/0	0/0	3/7/0	1/3/0	2/4/0
Radiation therapy (cur/pre/no)	0/0/10	0/0/7	1/2/7	0/1/3	1/1/4
Chemotherapy (cur/pre/no)	0/0/10	0/0/7	0/1/9	0/0/4	0/1/5
Glucocorticoid treatment (y/n)	0/10	0/0/7	0/0/10	0/0/4	0/0/6
Pky	4(0–28)	17.5(4–35)	40(10–75)*	50(10–50)*	25(17–75)*
fev_1_	106(71–120)	97(82–107)	76.5(47–113)*	78(47–113)*	76(67–90)*
fev_1_/fvc	80(70–88)	84(72–87)	63(49–69)*	62(50–67)*	64(49–69)*

Data are presented as number, or median and data range. COPD: chronic obstructive pulmonary disease; Smoking history: cur: current, ex: ex-smokers, no: never smoked; Lung Cancer: presence of lung cancer; NSCLC: Non-small cell lung cancer; SCLC: Small cell lung cancer; Type of NSCLC: adeno: adenocarcinoma, squa: squamous cell carcinoma, larg: large cell carcinoma; Radiation therapy: cur: current, pre: previous exposure, no: never exposed; Chemotherapy: cur: current, pre: previous exposure, no: never exposed; Pky: smoking pack years;fev_1_: forced expiratory volume in one second, % pred: percentage of predicted; fvc: forced vital capacity.

*: significant difference from control group, p<0.05. Note that one COPD patient was classified as ‘mild COPD’, with a fev_1_/fvc of 67.

### Bronchoalveolar lavage (BAL) and isolation of macrophages

Bronchoscopy was performed according to American Thoracic Society recommendations for the performance of bronchoscopy for investigative purposes, as previously described [25]. BAL was collected then centrifuged at 1600 RPM for 5 min and the supernatant frozen for later use. Macrophages were isolated by adhering to plastic as previously described, concentration adjusted to 4×10(5) cells/ml [3] then either used for phagocytosis or frozen immediately at -80°C for gene expression analysis.

### Cell culture and stimulation

THP-1monocytes (ATCC, Manassas, VA, USA) were differentiated into macrophages by seeding the cells at a density of 4×10(5) cells/mL in 24 well plastic plates, then stimulating with 100nM phorbol 12-myristate 13-acetate [26] in RPMI 1640 medium at 37°C with 5% CO2 for 72 has previously described [27]. Experiments were carried out between passages 6 and 20.

Exposure of THP-1 macrophages to cigarette smoke extract was carried as previously described [4,5,28,29]. After cigarette smoke extract exposure, non-adherent cells were discarded, and adherent cells were incubated with 500μL cold PBS for 5 min, then harvested by scraping the adherent cells with a plastic scraper into a 1.5 mL tubes prior to centrifugation at 4°C at 1600 RPM. Supernatant was removed and cell pellet stored at– 80°C for mRNA expression analysis.

### Reverse transcription and quantitative real-time PCR (qPCR)

The total RNA was extracted from snap frozen alveolar macrophages or THP-1 macrophages using the RNeasy Mini Kit (Qiagen, Doncaster, Australia) according to the manufacturer’s instructions. The concentration of total RNA was determined using standard photospectometry. The RNA quality expressed as RIN number was determined using the RNA Pico-Chip and Agilent 2100 Bioanalyzer (Agilent Technologies, Forest Hill, Australia). Complementary DNA (cDNA) was synthesized using RT^2^ HT First Strand Kit (Qiagen). Synthesized cDNA was stored at −20°C. The qPCR analyses of the samples were performed using Custom RT2 Profiler PCR arrays (Qiagen) and the ABI sequence detection system (StepOne Plus) as previously described [15], with modifications. Briefly, a 25μL reaction mixture into 96 well plates coated with primer pairs directed against components of the S1P system (*SPHK1*, *SPHK2*, S1P receptors (*S1PR1*, *S1PR2*, *S1PR3*, *S1PR4*, *S1PR5*) and the S1P-degrading enzymes *SGPP1*, *SGPP2* and *SGPL1*. Three reference genes, glucose 6-phosphate dehydrogenase (*G6PD*), hypoxanthine phosphoribosyl transferase 1 (*HPRT1*) and ribosomal protein L13a (*RPL13A*) were used in the array for data normalization. The efficiency and reliability of the PCR reaction was assessed by determining the efficiency of the reverse transcriptase reaction by Reverse-transcription control (RTC) determining, the efficiency of polymerase chain reaction itself by positive PCR control (PPC), and DNA contamination in the reaction by genomic DNA control (HGDC).

### Functional associations between sphingosine signalling system components and alveolar macrophage phagocytic ability in COPD

Phagocytosis of apoptotic cells was measured as previously reported [3,4,30]. Briefly, 16HBE bronchial epithelial cell targets were maintained in continuous culture, induced to apoptosis using UV, then labelled with sytox orange (Molecular Probes, Oregon, USA). The apoptotic cells were incubated with macrophages at a ratio of 10:1 for 1.5h. Non-adhered cells were removed and macrophages removed by gentle pipetting, following 5min incubation with 500uL ice-cold phosphate buffered saline (PBS). Macrophages that had ingested apoptotic cells were stained with CD13 phycoerythrin cyanine-7 (PE-Cy7) (BD Biosciences, San Jose CA, USA)), autofluorescence was quenched with trypan blue and 30,000 total events per tube were acquired immediately using a FACSCanto II Flow Cytometer (BD Biosciences). Macrophages were identified based on autofluorescence properties and staining with CD13. The percentage of the macrophages ingesting apoptotic cells was recorded.

To further elucidate functional effect of increased *S1PR5* on macrophage phagocytic ability, we performed the phagocytosis assay in the presence of varying concentrations of Suramin (Sigma Aldrich, Castle Hill, NSW, Australia), an antagonist of *S1PR3* and *S1PR5* [31]. Suramin at concentrations of 10nM to 10μM was added for 30min prior to the phagocytosis assay.

### Statistical analysis

Analysis was performed using SPSS statistic software (SPSS Inc. IBM Chicago, USA) and the two-sample Wilcoxon rank sum test, or the Kruskal-Wallis test for analyses of more than two groups. Correlation with lung function, smoking, age, gender and presence of lung cancer on mRNA expression levels of *SPHK*/S1P system genes were determined using data from all subjects and Pearson’s correlation coefficient with significance set at *p<0*.*05*.

## Results

### Characterization of expression profile of the S1P system in alveolar macrophages in healthy subjects

**Fig 1:**
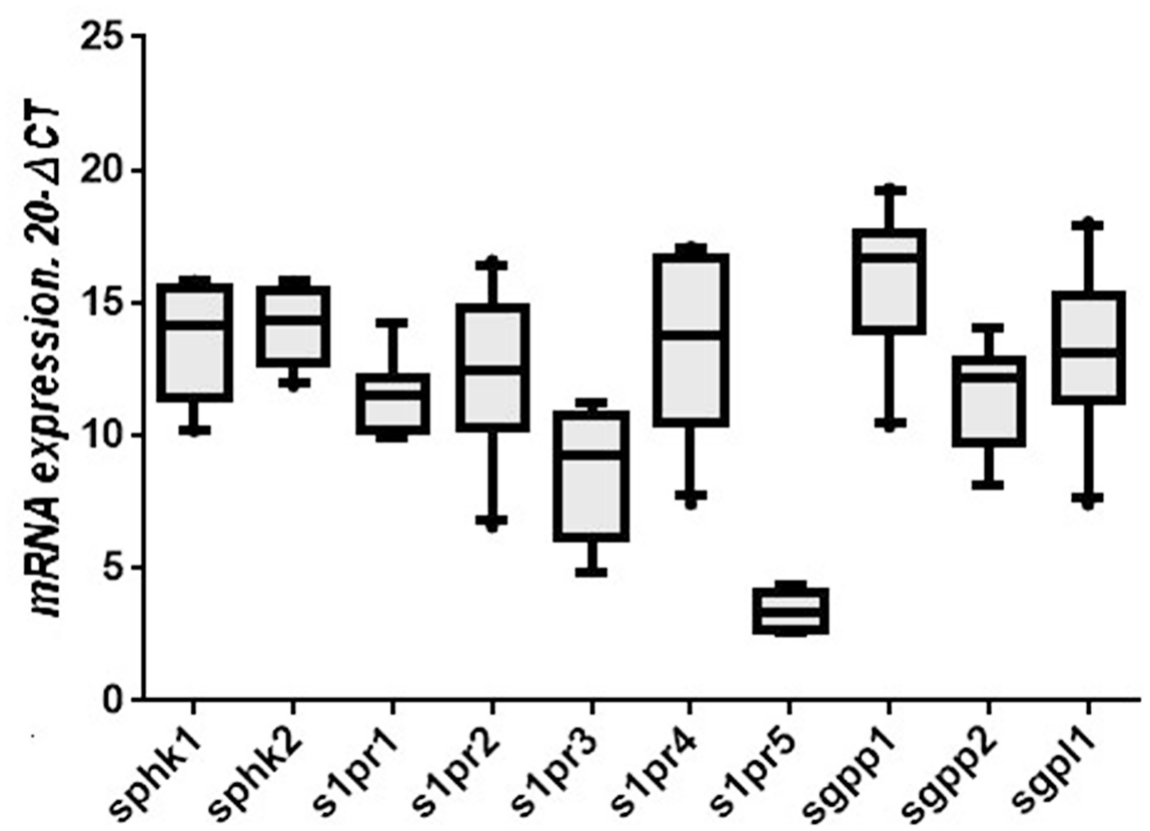
Relative mRNA expression levels of *SPHK*/S1P system genes in alveolar macrophages across normal subjectsc (N = 10). Box plots present median ± 25th and 75th percentiles (solid box) with the 10th and 90th percentiles shown by whiskers outside the box. The Ct values were subtracted from 20, so that higher values represent higher mRNA expression levels.

The mRNA expression levels of the components of the S1P system in alveolar macrophages from healthy individuals are unknown. We therefore compared the relative mRNA expression levels of the *SPHK*/S1P system genes in alveolar macrophages obtained from healthy control subjects (n = 10, Fig 1). All components of the S1P system were expressed in alveolar macrophages. We found no significant difference in the relative mRNA expression levels between the S1P synthesizing enzymes, *SPHK1* and*2*. In contrast, the S1P degradation enzymes, *SGPP1*, *SGPP2* and *SPGL1* were differentially expressed. *SGPP1* showed the highest, while SGPP2 had the lowest relative expression level. There were large differences in relative-mRNA expression levels among S1P receptors, with *S1PR4* being the predominant subtype while *S1PR5* showed the lowest expression level. The rank order of mRNA expression levels of all receptors from the high to low was *S1PR4*>*S1PR2*>*S1PR1*>*S1PR3*>*S1PR5*.

### Characterization of expression profile of the sphingosine system in alveolar macrophages in COPD

**Fig 2:**
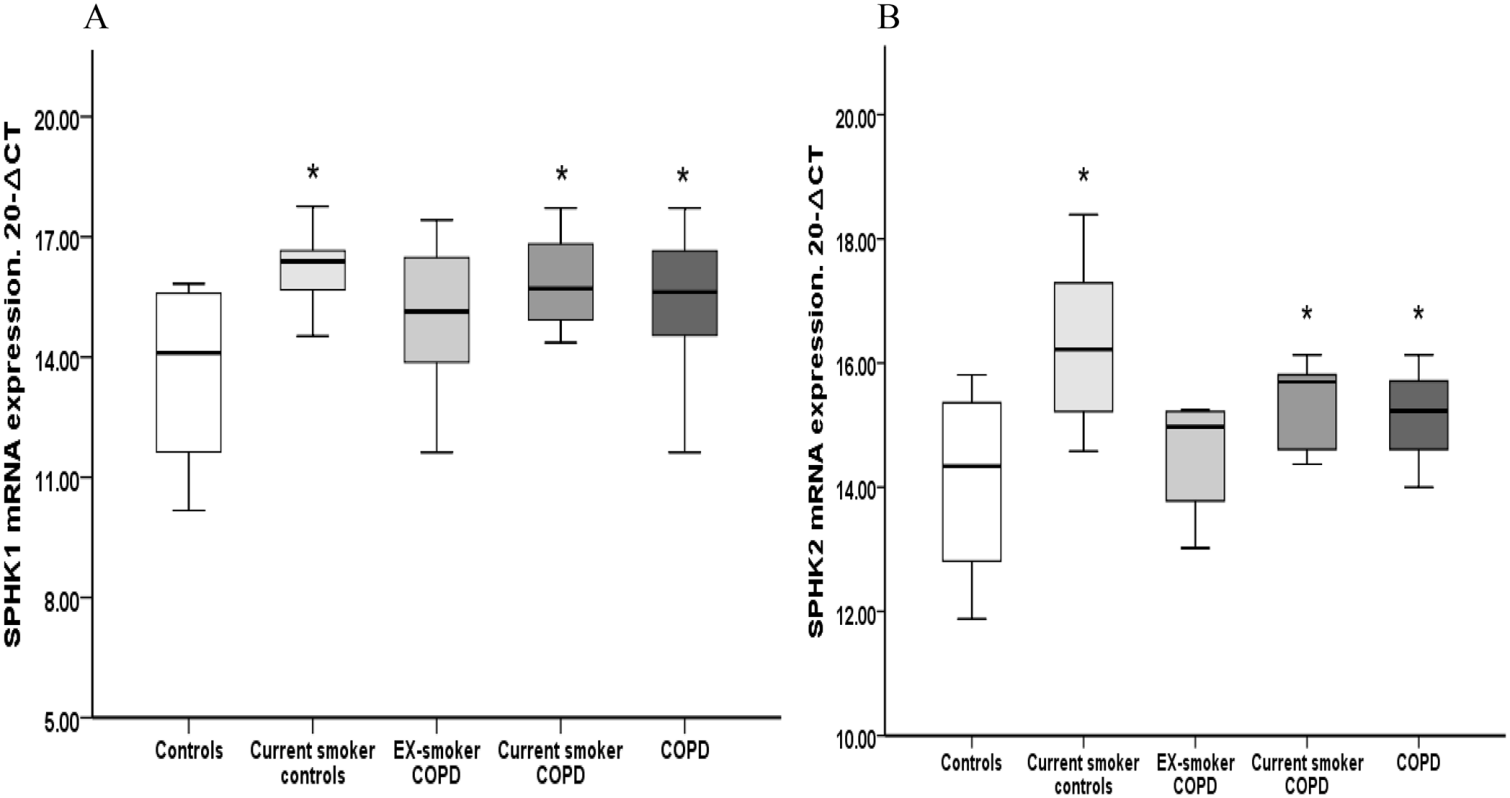
Relative mRNA expression of the S1P synthesizing enzymes, *SPHK1*, and *SPHK2* in alveolar macrophages. **(A)** Significantly higher mRNA expression of *SPHK1* was noted in current healthy smokers (6-fold increase), current smoker COPD (4.8-fold increase) and total COPD (3.5-fold increase) compared to control subjects (*p<0.05 vs control). No significant increase of *SPHK1* was found in alveolar macrophages isolated from ex-smoker COPD patients.** (B)** Significantly higher mRNA expression of *SPHK2* in current healthy smokers (4.4-fold increase), current smoker COPD(2.3-fold increase) and in the total COPD group (2.1-fold increase) versus control subjects (*p<0.05 vs control) was found. No significant increase in *SPHK2* was found in ex-smoker COPD subjects compared to control subjects. Data presented as box plots as described in Fig 1. The Ct values were subtracted from 20 so that higher values mean higher mRNA expression levels. Data presented as box plots as described in Fig 1. The Ct values were subtracted from 20 so that higher values represent higher mRNA expression levels.

There was significantly higher mRNA-expression of both *SPHK1*&*SPHK2* in alveolar macrophages from COPD patients (3.5 and 2.1fold increase respectively) compared to healthy controls (Fig 2). To assess the effect of smoking, the expression of *SPHK* was assessed in alveolar macrophages of healthy current smokers and current- or ex- smoker COPD patients. Compared to non-smoking individuals, a significantly higher relative mRNA expression of *SPHK1* was found in current healthy smokers (6-fold increase) and current-smoker COPD patients (4.8-fold increase) compared to control subjects (Fig 2A). Significantly higher mRNA expression levels for *SPHK2* were also found in current healthy smokers (4.4-fold increase) and current-smoker COPD subjects (2.3-fold increase) (Fig 2B). No significant changes in *SPHK1* or *SPHK2* expression were found in ex-smoker COPD patients, consistent with an effect of smoke rather than COPD disease on the relative expression levels of this *SPHK* isoform in alveolar macrophages.

**Fig 3:**
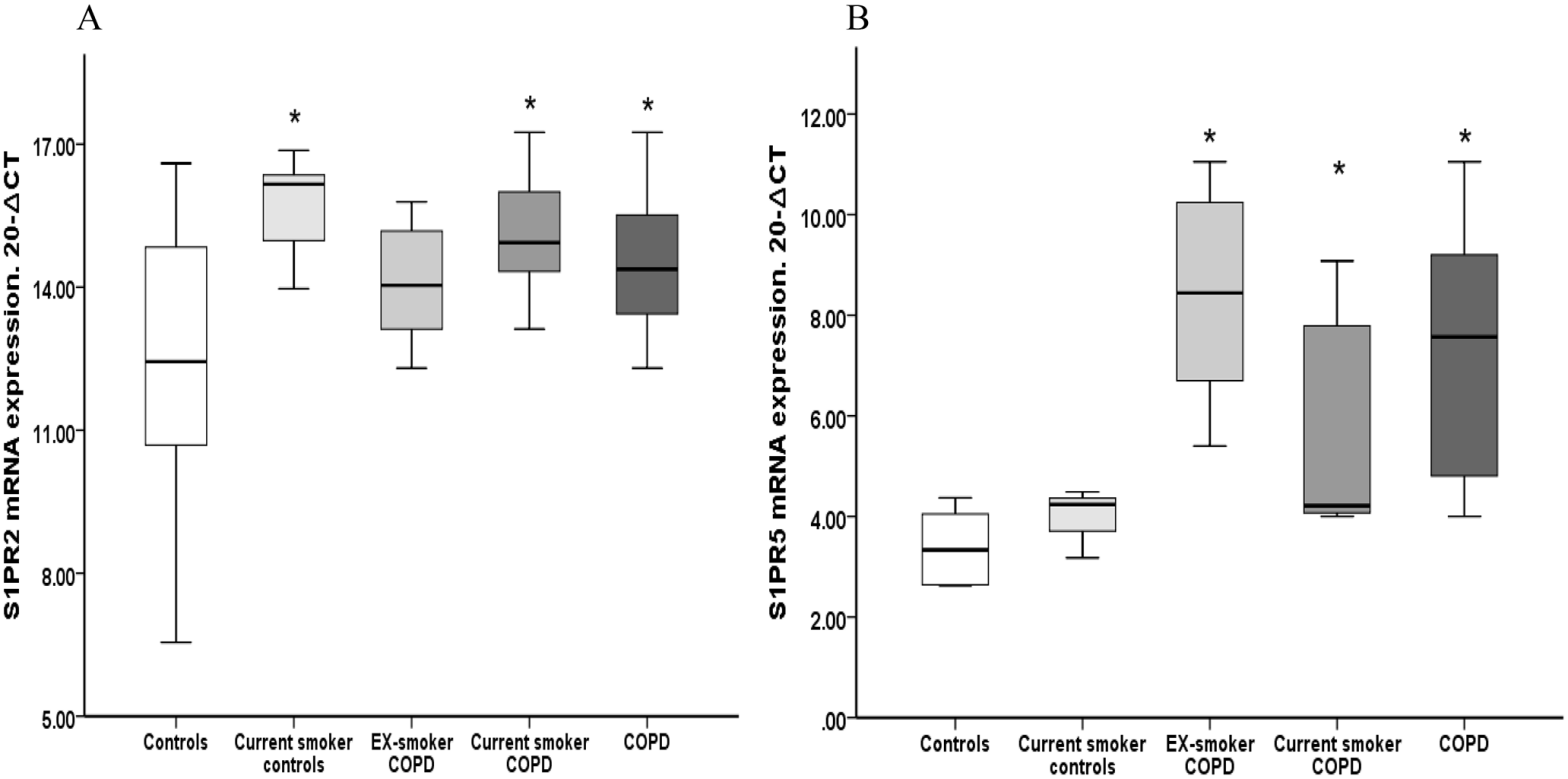
Relative mRNA expression levels of *S1PR2* and *S1PR5* in alveolar macrophages. (A) Significantly higher mRNA expression levels of *S1PR2* in current healthy smokers (9.3-fold increase), current smoker COPD subjects (6.1-fold increase) and in the total COPD group (4.3-fold increase) versus control subjects (*p<0.05 vs control) was found. No significant increase of *S1PR2* was found in ex-smoker COPD patients compared to control subjects.** (B)** Significantly higher mRNA expression of *S1PR5* was noted in in ex-smoker COPD subjects (32-fold increase), current smoker COPD subjects (5.4-fold increase) and in the COPD group (14.6-fold increase) versus healthy control subjects (*p<0.05 vs control). Data presented as box plots as described in Fig 1. The Ct values were subtracted from 20 so that higher values mean higher mRNA expression levels.

Significantly higher mRNA expression levels for *S1PR2* and *S1PR5* were present in alveolar macrophages from COPD subjects (4.3 and 14.6 fold increase respectively) compared to healthy controls (Fig 3). No significant differences were found between S1PR1, S1PR3 and S1PR4 and healthy controls (data not shown).

To assess the effect of smoking, the expression of S1P receptors was assessed in alveolar macrophages from healthy current smokers and current- or ex- smoker COPD patients. We found significantly higher mRNA expression for *S1PR2* in the healthy current smokers (9.3-fold increase p<0.05 vs control) and current-smoker COPD patients (6.1-fold increase p<0.05 vs control) compared to healthy controls (Fig 3A). Interestingly, no difference was found between ex-smoker COPD subjects and control subjects. In contrast, we observed significantly higher *S1PR5* mRNA levels in alveolar macrophages from both current- and ex-smoker COPD patients compared to control subjects (n = 10, p<0.05 vs control, Fig 3B). The basal very low *S1PR5* expression was 32-fold higher in alveolar macrophages from ex-smoker COPD patients and 5.4-fold higher from current-smoker COPD subjects compared to health controls. In addition there was no difference in the relative *S1PR5* expression levels between healthy smokers and non-smokers (Fig 3B), suggesting a COPD disease-specific effect rather than a cigarette smoke effect. No difference was found in *S1PR1*, *S1PR3* and *S1PR4* mRNA expression in healthy current smokers and current- or ex- smoker COPD patients compared to healthy controls (data not shown).

**Fig 4:**
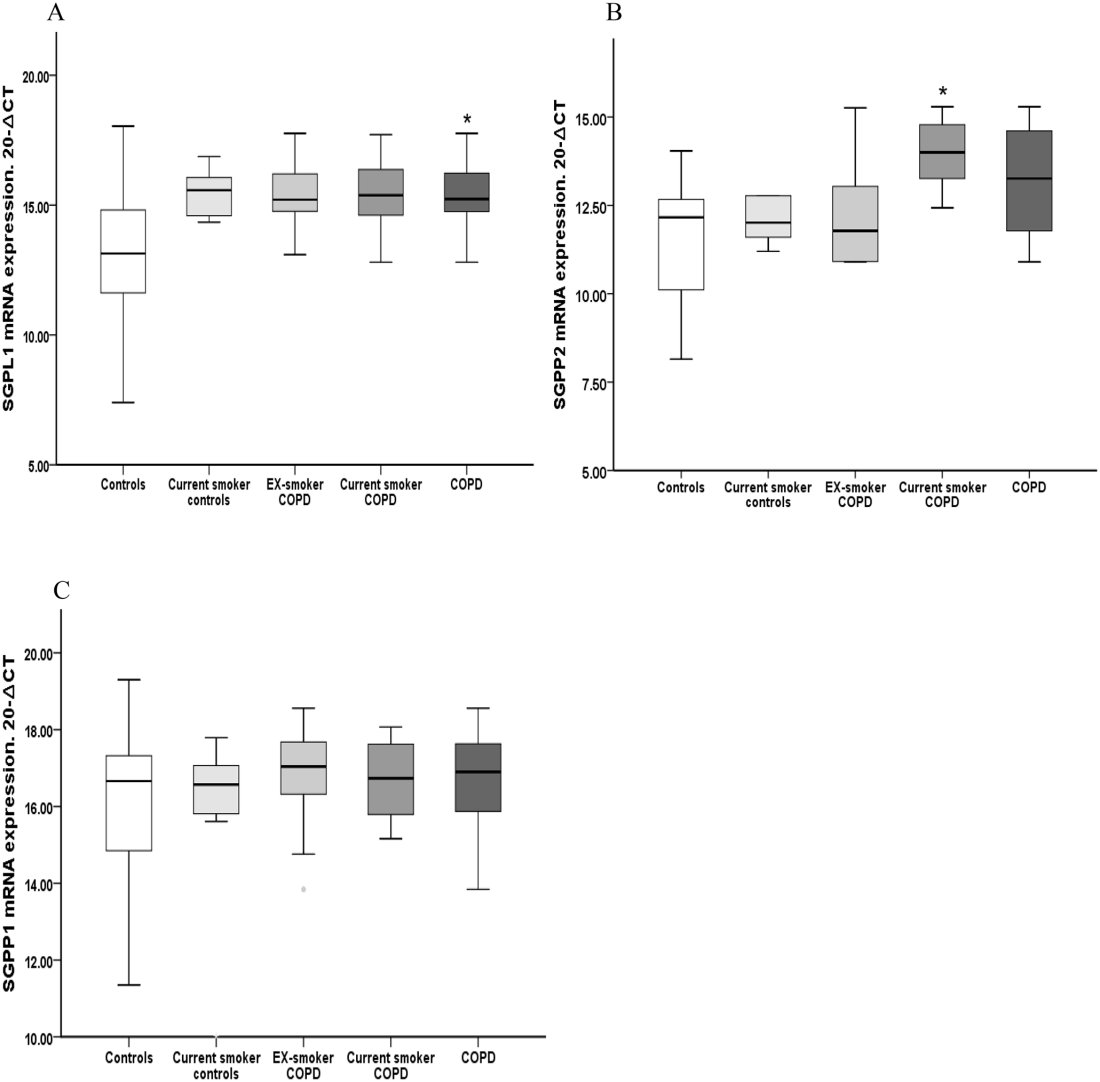
Relative mRNA expression of S1P degradation enzymes in alveolar macrophages. **(A)** Significantly higher mRNA expression of *SGPL1* in COPD patients (4.5-fold increase) versus control subjects (*p<0.05 vs control). No significant increase of *SGPL1*was found in current smoker COPD, ex-smoker COPD or healthy current smokers.** (B)** Significantly higher mRNA expression levels of *SGPP2* in current smoker COPD patients (6.1-fold increase) versus control subjects (*p<0.05 vs control) was noted. No significant increase of *SGPP2* was found in ex-smoker COPD subjects or healthy current smokers Data presented as box plots as described in Fig 1. The Ct values were subtracted from 20 so that higher values mean higher mRNA expression levels.

Significantly higher mRNA expression of *SGPL1* was found in COPD alveolar macrophages compared to cells from control subjects (4.5-fold increase p<0.05 COPD vs. control, Fig 4A). No significant differences were found between groups for either *SGPP1* or *SGPP2* (Fig 4B and 4C. However when we split the COPD on the basis of their smoking status we found significantly higher expression of *SGPP2* mRNA in alveolar macrophages from current-smoker COPD subjects compared to healthy control subjects (6.1-fold increase p<0.05 vs control, Fig 4). In contrast, no significant differences were found in *SGPP2* expression in either the ex-smoker COPD group or, healthy current smokers vs. healthy non-smoking controls.

A non-significant trend toward higher expression of *SGPL1* mRNA was found in healthy current smokers (p = 0.08) and in both ex-smoker and current-smoker COPD subjects (p = 0.06 for both) compared to control subjects. No significant differences were found in relative mRNA expression for *SGPP1* between healthy current smokers, ex-smoker COPD subjects and current smoker COPD subjects compared to control subjects (Data not shown).

### Functional associations between S1P signalling system components and alveolar macrophage phagocytic ability in COPD

**Fig 5:**
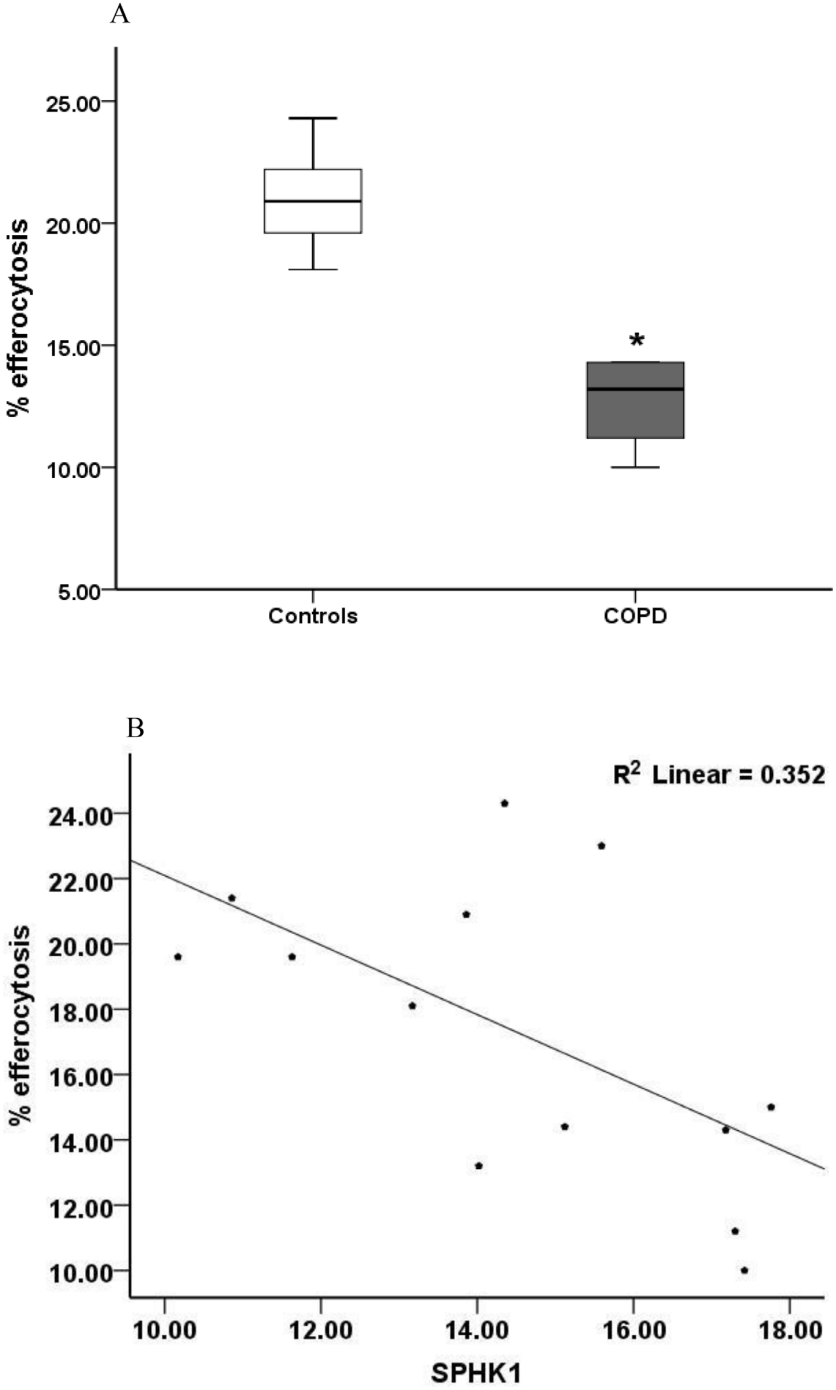
**(A) Phagocytosis of apoptotic 16HBE airway epithelial cells (AEC) by alveolar macrophages from chronic obstructive pulmonary disease (COPD) subjects and controls.** Compared to alveolar macrophages from healthy controls, macrophages from COPD patients had a significantly reduced ability to phagocytose apoptotic 16HBE epithelial cells (*p<0.05 vs control). **(B) Correlation between the relative mRNA expression of SPHK1 and % efferocytosis.** There was a significant correlation between efferocytosis and mRNA expression of *SPHK1* (p<0.05). The correlation coefficients are listed in the results.

To assess the functional relevance of our findings with regard to macrophage phagocytic function we investigated the association between phagocytosis and expression pattern of the S1P signalling system components. Consistent with our previous reports [3–7], a significantly reduced ability to phagocytose apoptotic cells was observed in alveolar macrophages from COPD patients in comparison to healthy control macrophages (mean±SEM, COPD 12.62±2.43% vs controls 20.98±1.98% p = 0.004, Fig 5A. For 13 COPD subjects, a strong negative correlation was found between *SPHK1*, *S1PR3* and *S1PR5* mRNA expression levels and the ability of alveolar macrophages to phagocytose apoptotic cells (*SPHK1* r = -0.59, p<0.05; *S1PR3* r = -0.70, p<0.05; *S1PR5* r = -0.87 p<0.05; *SPHK1* data presented in Fig 5B. No significant associations were found between the presence of lung cancer and macrophage phagocytic ability (r = -0.471, p = 0.098).

**Fig 6:**
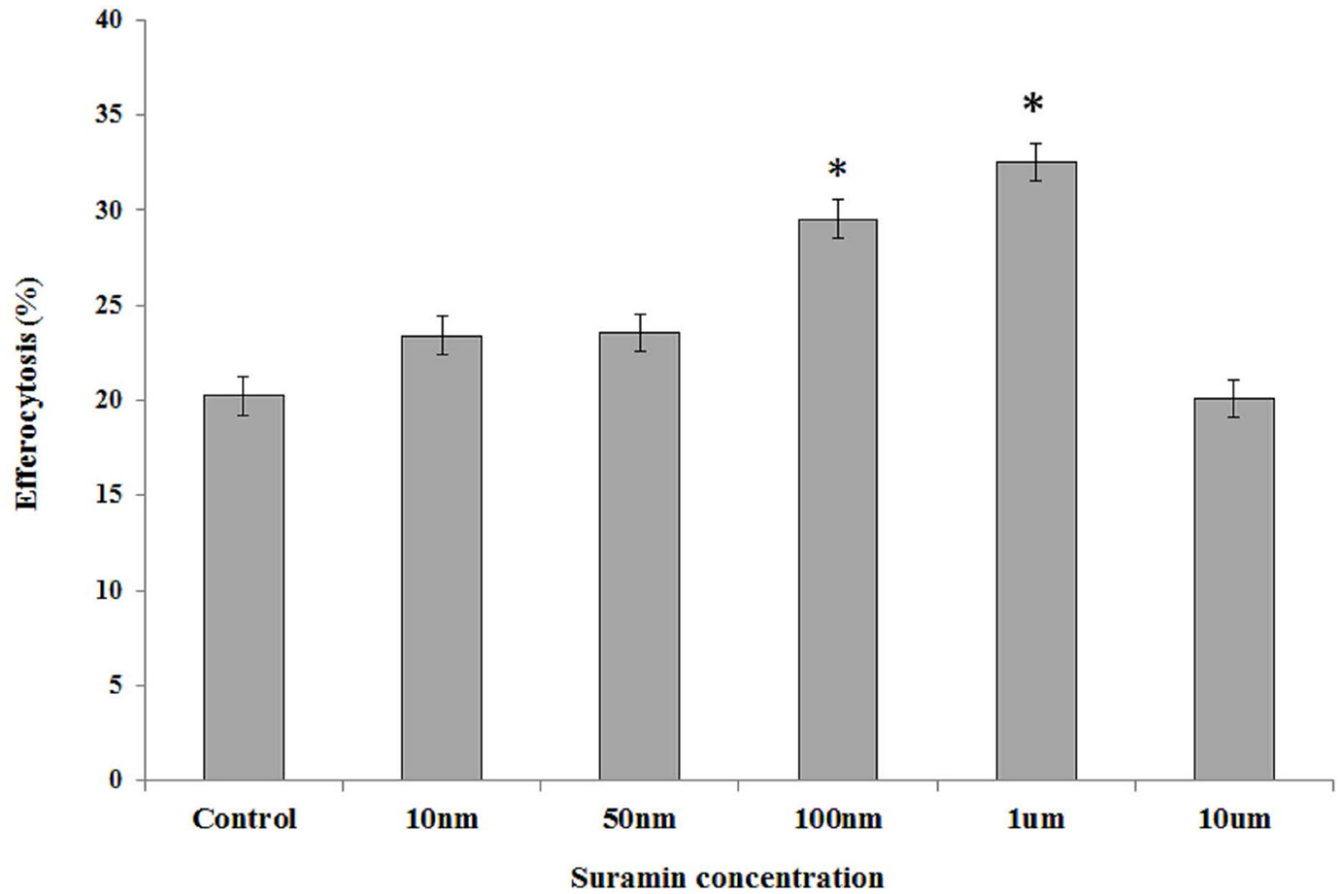
Effects of antagonising *S1PR3* and *S1PR5* with Suramin. To further elucidate functional effect of increased *S1PR5* on macrophage phagocytic ability, we performed the phagocytosis assay in the presence of varying concentrations of Suramin, an antagonist of *S1PR3* and *S1PR5*. Suramin at concentrations of 10nM to 10μM were added for 30 min prior to the phagocytosis assay. We noted a significant (p<0.05) increase in phagocytosis of apoptotic cells in the presence of Suramin.

Phagocytosis of apoptotic cells was dose dependently increased by the addition of the *S1PR3* and *S1PR5* agonist Suramin, with a maximum effect at a concentration of 1uM (Fig 6).

### Associations between S1P signalling system components in alveolar macrophages

**Fig 7:**
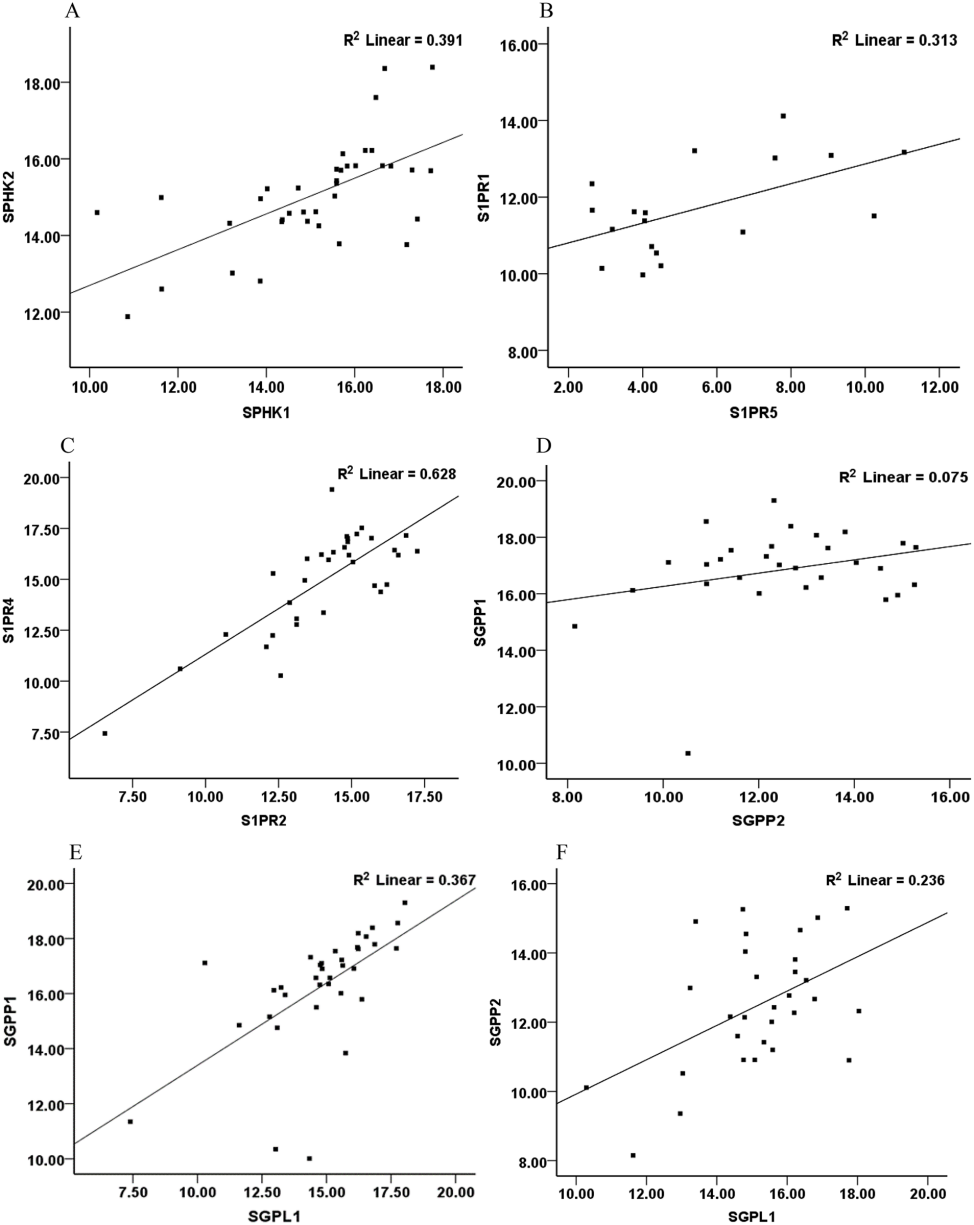
Correlations between relative mRNA expression of various components of the S1P signalling system in alveolar macrophages. **(A)** correlation between *SPHK1 and* 2, p<0.05; **(B)** correlation between *S1PR1 and S1PR5*, p<0.05; **(C)** correlation between *S1PR2 and S1PR4*, p<0.05; **(D)** correlation between *SGPP1 and SGPP2* p<0.05; **(E)** correlation between *SGPL1* and *SGPP1* p<0.05; **(F)** *SGPL1* and *SGPP2*. The correlation coefficients are listed in the results.

Since the components of the *SPHK*/S1P system are part of a combined signalling system, we analysed correlations between expressions of the various components. A strong positive correlation between the relative mRNA expression levels was observed for *SPHK1* and *SPHK2* (r = 0.62, p<0. 05) (Fig 7A. In addition a strong positive correlation was found between S1P receptor subtypes e.g. *S1PR1* and *S1PR5* (r = 0.55, p<0.05); *S1PR2* and *S1PR4* (r = 0.79, p<0.05; Fig 7B & 7C. We also noted a strong positive correlation between *SPHK1* and *S1PR2* (r = 0.81, p<0.05). There were positive correlations between the S1P degradation enzymes; *SGPP1* significantly correlated with *SGPP2* (r = 0.38, p<0.05) (Fig 7D; *SGPL1* significantly correlated with both *SGPP1* and *SGPP2* (r = 0.60, p<0.05 and r = 0.54, p<0.05 respectively; Fig 7E & 7F).

### Associations between S1P signalling system components and fev_1_, fev_1_/fvc, smoking status, pack years, age, gender, presence of cancer, type of cancer and previous or ongoing chemotherapy and/or radiotherapy

**Fig 8:**
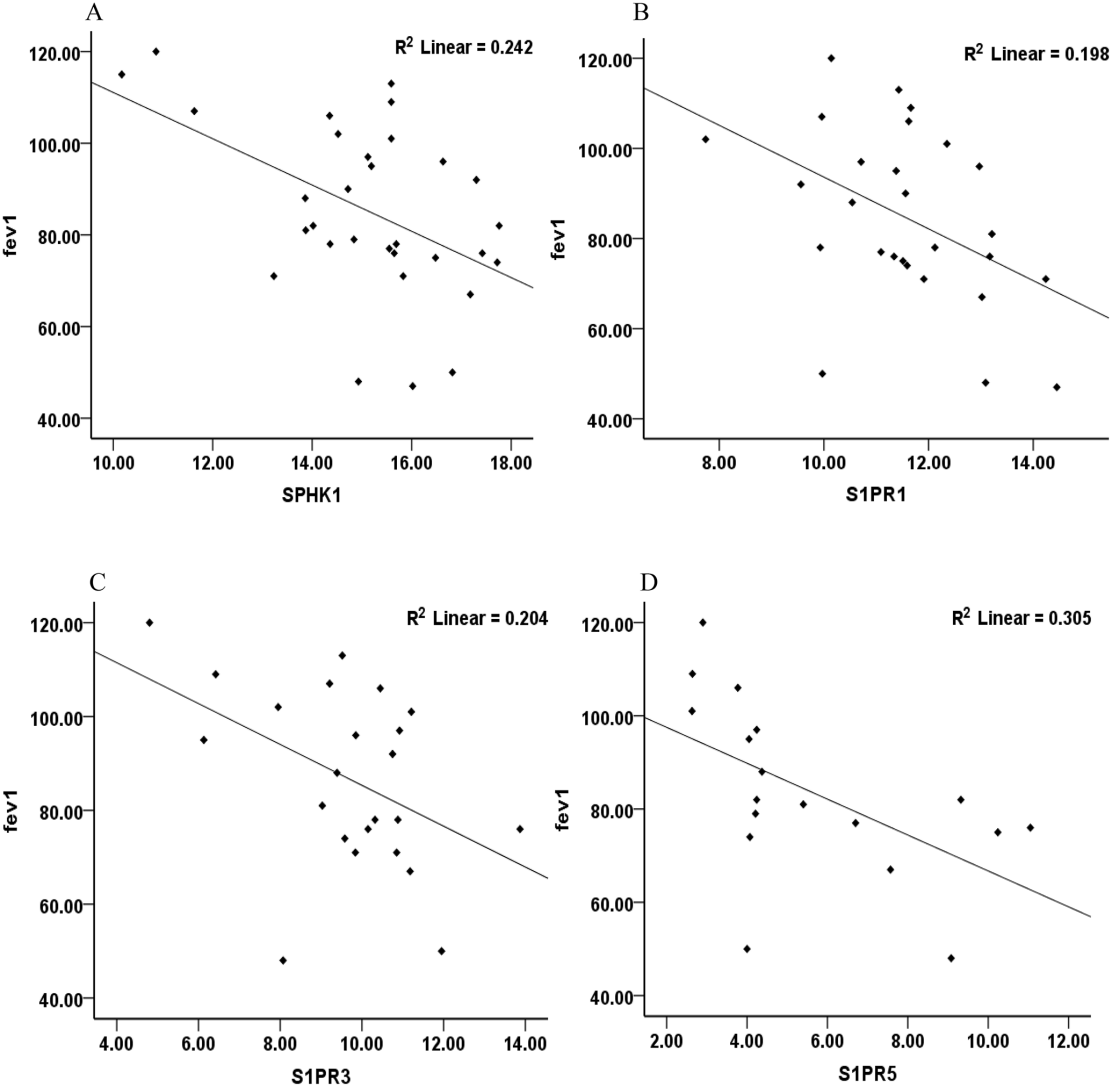
Correlations between the relative mRNA expression of various components of the S1P signalling system and lung function (fev_1_). Significant (p<0.05) correlations were found between fev_1_ and **(A)** *SPHK1* **(B)** *S1PR1* **(C)** *S1PR3*, **(D)** *S1PR5*, p<0.05. The correlation coefficients are listed in the results.

A strong negative correlation was found between lung function (fev_1_) and mRNA expression levels of *SPHK1*, *S1PR1*, *S1PR3* and *S1PR5* in alveolar macrophages (*SPHK1* r = -0.49, p<0.05; *S1PR1* r = -0.44 p<0.05; *S1PR3*, r = -0.45, p<0.05; *S1PR5* r = -0.55, p<0.05; Fig 8), with a trend observed for both *SGPL1* and *SGPP2* with fev_1_ (*SGPL1* r = -0.32, p = .078; *SGPP2* r = -0.37, p = .091; Fig 8). Furthermore a negative correlation was observed between the fev_1_/fvc ratio, a further indicator of lung function, and mRNA expression levels of *S1PR5* and *S1PR3* (*S1PR5* r = -0.61 p<0.05; *S1PR3* r = -0.52 p<0.05), with a trend observed between *SPHK1* and fev_1_/fvc (r = -0.33, p = .063).

We found a significant positive correlation between smoking status and relative mRNA expression levels of both *SPHK* isoforms, *S1PR2*, *S1PR4* and *SGPP2* (*SPHK1*r = 0.41, p<0.05; *SPHK2* r = 0.38, p<0.05; *S1PR2* r = 0.36, p<0.05, *S1PR4* r = 0.35, p<0.05; *SGPP2* r = 0.39, p<0.05). There were significant positive correlations between age and mRNA expression levels of *S1PR1* and *S1PR3* (*S1PR1* r = 0.39, p<0.05; *S1PR3* r = 0.45, p<0.05). Moreover a high positive correlation was revealed between mRNA expression levels of *SPHK2* and *S1PR5* and smoking pack years (*SPHK2* r = 0.36, p<0. 05; *S1PR5* r = 0.54, p<0. 05). No correlation was found between gender and any of the S1P system mRNA levels. The only significant correlation between the presence of cancer and S1P system mRNA expression levels was for *S1PR3* that showed a positive correlation with the presence of cancer independent of COPD status(r = 0.41, p<0.05). Moreover, no significant associations were found between S1P system mRNA expression levels with types of lung cancer or exposure to chemotherapy and/or radiotherapy.

### Investigation of the effect of cigarette smoke extract on the expression of *SPHK1*, *SPHK2*, *S1PR2*, *S1PR5* and *SGPP2* in THP-1 macrophages *in vitro*

**Fig 9:**
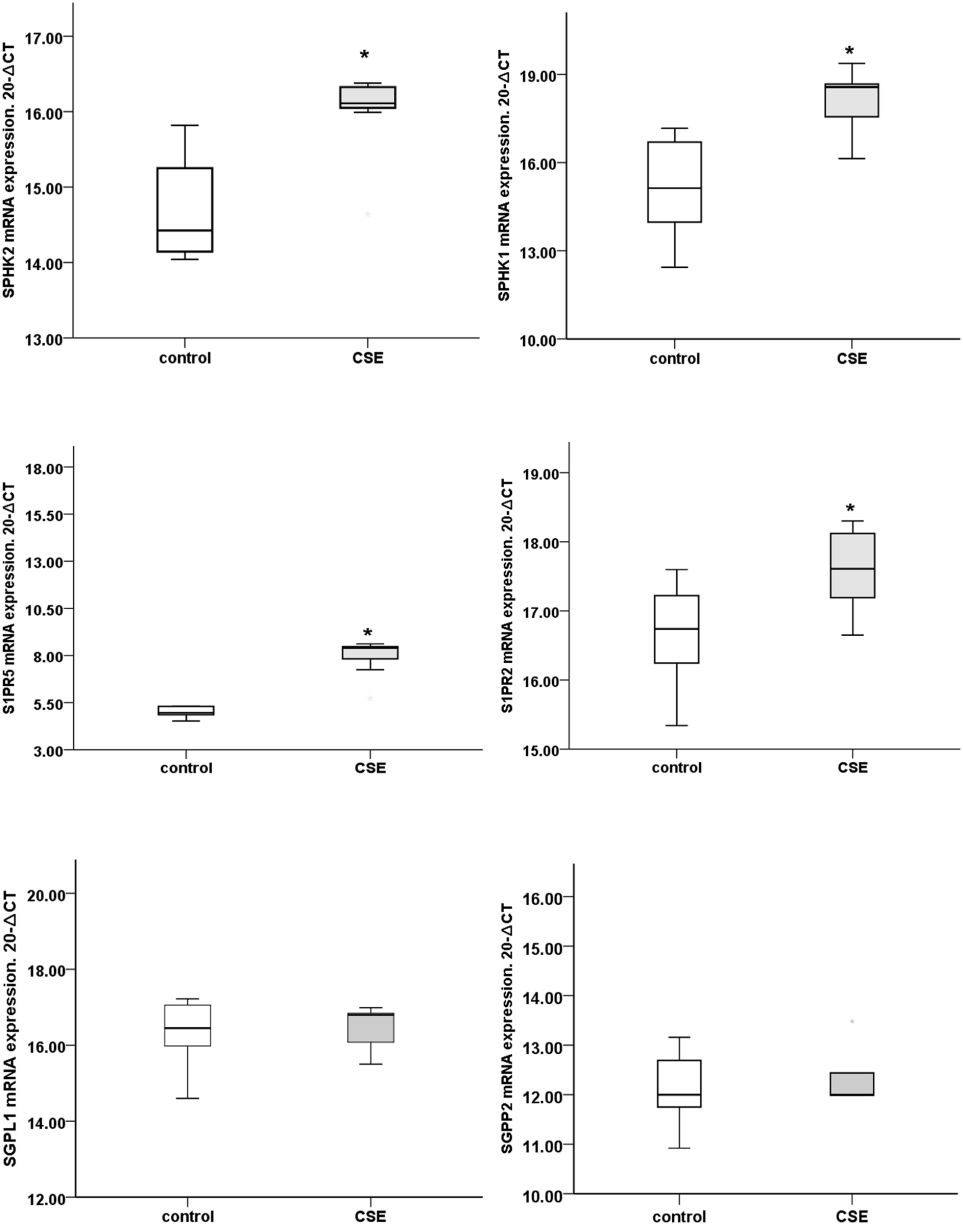
mRNA expression of various components of the S1P signalling system in THP-1 macrophages following stimulation with 10% cigarette smoke extract for 24h. *:Significant increase in mRNA expression of *SPHK1*, *SPHK2*,*S1PR2*, *S1PR5*, but no significant changes were found in *SGPL1* and *SGPP2* compared to non-stimulated macrophages (p<0.05).

Assessment of S1P system genes showed variation according to their passages, with expression pattern comparable to normal human alveolar macrophages between passages 6 to 20. To confirm the effects of cigarette smoke on macrophage expression of *SPHK1*, *SPHK2*, *S1PR2*, *S1PR5* and *SGPP2* we determined the expression levels of the *SPHK*/S1P system in THP-1 macrophages exposed to 10% cigarette smoke extract for 24h. Consistent with the alveolar macrophages from human smoker COPD subjects, we noted significantly higher relative mRNA expression of *SPHK1*, *SPHK2*, *S1PR5* and *S1PR2* whereas there was no increase in expression of *SGPP2* and *SGPL1* (n = 7experiments p<0.05, Fig 9).

## Discussion

In recent years it has become evident that alveolar macrophages play a vital role in the pathophysiology and development of COPD [31]. Macrophages modulate and control levels of inflammation; however, the macrophage-dependent inflammatory haemostasis is defective in COPD [31]. One of the major causes of this defect is thought to be a reduced ability of alveolar macrophages to phagocytose apoptotic cells and bacteria [3,32] which contributes to ongoing inflammation in lungs of these patients [8,33]. Several studies have reported changes in candidate molecules that may at least partially contribute to the defective efferocytosis in COPD, although it is becoming clear that a more complex network of molecules rather than a single molecule is the more likely cause of the reduced efferocytosis [34,35]. In particular, identification of molecule(s) that are affected in COPD irrespective of smoking status would be an important strategy toward identification of new macrophage-targeted treatments for COPD.

Previous studies have identified the S1P system as a candidate for macrophage dysfunction. McQuiston et al. showed that the S1P-mediated increase in phagocytosis occurred in an antibody-dependent manner [23,36]. The antibody-mediated pathway is commonly linked to phagocytosis of pathogen; however, it became clear in the last decade that apoptotic cells can also become opsonized with naturally occurring IgM auto antibodies which facilitate their internalization [37–40] This mechanism is particularly efficient when combined with the complement [40]. Petrusca et al. reported that up-regulation of the S1P precursor ceramide inhibited the alveolar macrophage-mediated clearance of apoptotic cells [24]. A further study on murine microglia, resident macrophage-like cells of the CNS which are involved in phagocytosis and inflammatory responses, showed the involvement of *SPHK1* signalling in their functions [41]. These studies, however, were focused on single components of the S1P system, and to the best of our knowledge, there is no study that has comprehensively studied the combination of synthesizing, degrading enzymes and receptors in human alveolar macrophages in COPD.

S1P, the product of SPHK1 and 2 has been implicated in many biological functions in the cell, including survival, proliferation and migration, and macrophage phagocytic function [16,20,42,43]. In the present study, the expression of both *SPHK1* and *SPHK2* mRNAs was significantly increased in alveolar macrophages from healthy smokers and current-smoker COPD subjects, with no changes found in macrophages obtained from ex-smoker COPD subjects. This indicates a close association between smoking and expression of S1P synthesizing enzymes. The effect of cigarette smoke was further highlighted by a significant increase in *SPHK1* in the model of cigarette smoke-exposed THP-1 macrophages. Furthermore analysis of expression and patient data showed and a positive correlation between *SPHK1* and smoking status. Smoking causes inflammation and inflammation is known to modulate *SPHK1* mRNA expression in animal macrophages [41,44,45]. In this study we showed for the first time the response in *SPHK1* in the context of COPD or cigarette smoking. Increased ceramide levels in alveolar macrophages have been shown to impair their ability to phagocytose apoptotic cells [24]. Ceramide is converted to sphingosine which is phosphorylated via *SPHK1* and *SPHK2* to S1P [46]. It is thus possible that the higher mRNA expression levels of *SPHK1* or *SPHK2* in alveolar macrophages in response to smoking reflect the response to high ceramide levels with the aim to reduce ceramide concentration.

A finely-tuned balance between *SPHK1* and *SPHK2* expression and functions, with tissue specificity has been reported [47]. For example, in rodent neuronal tissue there are higher levels of *SPHK2* compared to *SPHK1* [48], whereas in murine lungs there is higher expression and activity of *SPHK1* [49,50]. It should be noted that the expression in the lung reflects many cell types including endothelial, epithelial cells and macrophages. Interestingly in the present study, we observed equal relative mRNA-expression levels for *SPHK1* and *SPHK2*, and a correlation between the two in alveolar macrophages from healthy controls. In contrast, the two kinases were differentially expressed in alveolar macrophages from COPD subjects and although both *SPHK1* and *SPHK2* separately correlated positively with smoking status, only *SPHK1* was found to be correlated with lung function suggesting a changed mRNA expression and presumably a change in balance between the kinases and the potential involvement of *SPHK1* in smoking-related COPD disease progression.

S1P receptors are essential for S1P function [19]. Changes in expression of these receptors have been reported in many cell types in various lung diseases including acute lung injury, asthma, cystic fibrosis and COPD [51]. The distribution of the receptors has been shown to be cell specific and affected differently in the various disease conditions [16]. The presence of S1P receptors in macrophages has been well documented [20,52]; however, the presence and expression in human alveolar macrophages is less studied. The *S1PR4* subtype showed the highest relative mRNA expression followed by the *S1PR2* subtype. Consistent with our data for *SPHK*s, we found that expression of *S1PR2* mRNA in alveolar macrophages increased with smoking, and increased in both healthy current smokers and current-smoker COPD subjects, but was unchanged in ex-smoker COPD subjects. In addition, our *in vitro* investigations revealed a significant increase in *S1PR2* in cigarette smoke extract exposed THP-1 macrophages, confirming the direct effects of cigarette smoke on expression of this receptor. A potential role for this receptor in phagocytosis was also shown by McQuiston et al who showed that treatment with the dual S1PR2/4 antagonist JTE-013 affected the phagocytosis of *Cryptococcus neoformans* by wild-type alveolar macrophages in contrast with the S1PR1 antagonists W146 or VPC 23901 [23,36]. In non-alveolar macrophages/monocytes activation of *S1PR2* attenuated migration [53]. This could relate to cigarette smoke-induced inhibition of macrophage function in alveoli. *S1PR2* may also play a role in the activation of cytoskeletal-remodelling that occurs during efferocytosis. The increased cigarette smoke-related *S1PR2* expression by alveolar macrophages found in our study was therefore surprising; however, we also noted a positive correlation between *S1PR2* and *S1PR4*. Efferocytosis occurs with extensive pseudopod extensions and is dependent on the relative activation of Rho GTPases, Rac-1 and RhoA [54] that control actin polymerisation and formation of lamellipodia that is required for macrophage engulfment of apoptotic cells. We and others have shown that Rho activity is increased and Rac-1 activity decreased, in macrophages in response to cigarette smoke [6,55,56]. Interestingly, expression of S1PR4 protein has also been shown to activate RhoA in response to S1P stimulation in Chinese hamster ovary (CHO) cells [57], and S1P has been shown to promote migration of cells expressing *S1PR4* through activation Cdc42, a Rho family member [58]. The positive correlation we found between *S1PR2* and *S1PR4* thus suggests that these receptors may potentially work in concert to negatively affect the efferocytosis process in response to cigarette smoke.

*S1PR2* expression also induces increased macrophage accumulation in atherosclerosis plaque and promotion of inflammation in mice [59,60], and its increased expression could thus also contribute to the macrophage accumulation that has been reported in current smokers with or without COPD [61,62].

In contrast to the findings with *SPHK1*, *SPHK2* and *S1PR2*, we found that *S1PR5* was higher in alveolar macrophages from both ex-smoker COPD and current smoker COPD subjects and negatively correlated with both fev_1_ and fev/fvc indicating a possible involvement in COPD progression that is at least to some extent independent of cigarette smoking. Unlike *S1PR2*, there were no correlations between *S1PR5* and smoking status, or with *SPHK1* or *SPHK2* or *S1PR2*. Currently, the role of *S1PR5* in macrophages is unclear, although our findings of a strong negative correlation between expression of *S1PR5* and phagocytosis of apoptotic cells suggest that it may play an important role in this function and contribute to the defective macrophage function in COPD. Several studies have shown that mRNA levels not always predict protein expression. We therefore utilized a functional approach to assert the increase of S1PR5 and its effects on macrophage phagocytic ability. We used a *S1PR3/5* antagonist suramin [31], and found that phagocytosis was dose dependently increased with a maximum effect at 1uM suramin. Taken together with our findings of significant associations between the increased expression of *SPHK*, *S1PR2* and *S1PR5*, and alveolar macrophage phagocytic ability in COPD, these data support a functional effect of the increased gene expression of *S1PR5*. *S1PR5* has been reported to interact with Rho-G_12/13_ protein coupled Rho/ROCK signalling pathway in oligodendrocyte precursor cells (OPCs) and appears to mediate their migration [63]. Although we did not find significantly increased expression of *S1PR1* in alveolar macrophages from either smokers or COPD subjects, this receptor was found to be strongly correlated with *S1PR5*. Taken together with our findings of a negative correlation with lung function and phagocytosis of apoptotic cells, these data suggest that both receptors may be associated with COPD progression and defective macrophage function in COPD either by a link between the two or common downstream effectors. Consistent with this, a previous study showed that both *S1PR1* and *S1PR5* enhanced microgliosis [64]. It is thus likely that the defective efferocytosis in COPD that persists despite smoke cessation could be mediated by *S1PR5*, possibly in association with *S1PR1*. Interestingly, in our previous study the relative mRNA for *S1PR5* was significantly lower in whole lung tissue of patients with COPD compared to controls without COPD [16]. In the present study, however, alveolar macrophage-specific S1PR5 expression was increased when comparing COPD vs. controls. This counterintuitive finding further highlights the finely tuned balance and the tissue specificity of the sphingosine family, and stresses the need to investigate not only whole lung tissue, but individual cell types of interest.

None of the controls in this study suffered from lung cancer whereas 60% of the COPD patients did. We therefore correlated S1P system mRNA expression levels with the presence of lung cancer. The only significant correlation however was for *S1PR3* that showed a positive correlation with the presence of cancer independent of COPD status, suggesting that the findings in COPD subjects in our study were not influenced by the presence of lung cancer. A potential limitation of the study is that we did not measure S1P levels in BALF as this would have provided an additional understanding of smoke- and/or COPD-related changes in S1P signalling.

S1P degradation enzymes, *SGPP1*, *SGPP2* and*SGPL1* are enzymes that metabolize S1P in the cell and their importance comes from their role in controlling the levels of S1P in the cell, excessive concentrations of which are detrimental to cell fate and functions [42,65] as well as drug resistance and cancer [66]. In the present study, *SGPL1* was found to be higher in alveolar macrophages from COPD subjects while *SGPP2* was higher in current-smoker COPD subjects compared to healthy controls, with a trend towards a significant correlation between *SGPP2* and lung function. In parallel, we also found that cigarette smoke failed to increase mRNA levels of *SGPP2* and *SGPL1* in our in vitro cell line experiments. Recently, SNP variants on *SGPP2* in lung epithelial cells from adult smoker subjects found to be associated with vitamin D regulations as well as and fev_1_ and fev/fvc ratio [67]. Furthermore, vitamin D-binding protein (DBP) levels were found to be directly responsible for alveolar macrophage activation as well as related to fev_1_ [68]. We also noted a non-significant trend for an increase in *SGPL1*, the other S1P degradation enzyme, in healthy smokers and both and current-and ex-smoker COPD subjects as well as a positive correlation with *SGPP2*, suggesting a link between these two enzymes in alveolar macrophages in COPD. *SGPP2* converts S1P to sphingosine which can be converted back to ceramide and *SGPL1* degrades S1P, which indirectly can favour an increase in ceramide over S1P, so the increase in both degradation enzymes is likely to impact on the increasing ceramide levels and subsequent defect in alveolar macrophage efferocytosis ability. In addition, the established link between COPD and vitamin D and alveolar macrophage function [69–71], and the role of these enzymes in degradation of S1P [18] suggests a potential important indirect role of *SGPP2* and/or *SGPL1* in the alveolar macrophage defect in COPD. Interestingly, a correlation between *SGPP1* and *SGPL1* expression by alveolar macrophages was found in the present study, further supporting a link between these two enzymes.

In conclusion, our data strongly support the role of the S1P signalling pathway in the defective alveolar macrophage function and resultant chronic inflammation that is present in the airways of patients with COPD. In particular, we identified *S1PR5* and S1P degradation enzymes as mediators that are independently increased in alveolar macrophages in COPD, independent of the presence of cigarette smoke and, importantly, a significant correlation between *S1PR5* mRNA expression and lung function. These mediators are thus worthy of their further investigation as macrophage-target therapeutic strategies for COPD and other chronic inflammatory lung diseases.
